# Risk factors and incidence over time for lower extremity amputations in people with type 1 diabetes: an observational cohort study of 46,088 patients from the Swedish National Diabetes Registry

**DOI:** 10.1007/s00125-021-05550-z

**Published:** 2021-09-08

**Authors:** Sara Hallström, Ann-Marie Svensson, Aldina Pivodic, Arndís F. Ólafsdóttir, Magnus Löndahl, Hans Wedel, Marcus Lind

**Affiliations:** 1 Department of Internal Medicine, Sahlgrenska University Hospital, Gothenburg, Sweden; 2 Department of Molecular and Clinical Medicine, University of Gothenburg, Gothenburg, Sweden; 3 Center of Registers in Region Västra Götaland, Gothenburg, Sweden; 4 Department of Clinical Neuroscience, Institute of Neuroscience and Physiology, Sahlgrenska Academy, University of Gothenburg, Gothenburg, Sweden; 5 Statistiska Konsultgruppen, Gothenburg, Sweden; 6 Department of Medicine, NU-Hospital Group, Uddevalla, Sweden; 7 Department of Clinical Sciences Lund, Faculty of Medicine, Lund University, Lund, Sweden; 8 Department of Endocrinology, Skane University Hospital, Lund, Sweden; 9 Department of Health Metrics, Sahlgrenska Academy, University of Gothenburg, Gothenburg, Sweden

**Keywords:** Amputation, Cardiovascular disease, Epidemiology, HbA_1c_, Lower-extremity amputation, Risk factors, Type 1 diabetes

## Abstract

**Aims/hypothesis:**

The aim of this work was to study the incidence over time of lower extremity amputations and determine variables associated with increased risk of amputations in people with type 1 diabetes.

**Methods:**

Individuals with type 1 diabetes registered in the Swedish National Diabetes Registry with no previous amputation from 1 January 1998 and followed to 2 October 2019 were included. Time-updated Cox regression and gradient of risk per SD were used to evaluate the impact of risk factors on the incidence of amputation. Age- and sex-adjusted incidences were estimated over time.

**Results:**

Of 46,088 people with type 1 diabetes with no previous amputation (mean age 32.5 years [SD 14.5], 25,354 [55%] male sex), 1519 (3.3%) underwent amputation. Median follow-up was 12.4 years. The standardised incidence for any amputation in 1998–2001 was 2.84 (95% CI 2.32, 3.36) per 1000 person-years and decreased to 1.64 (95% CI 1.38, 1.90) per 1000 person-years in 2017–2019. The incidence for minor and major amputations showed a similar pattern. Hyperglycaemia and renal dysfunction were the strongest risk factors for amputation, followed by older age, male sex, cardiovascular comorbidities, smoking and hypertension. Glycaemic control and age- and sex-adjusted renal function improved during the corresponding time period as amputations decreased.

**Conclusions/interpretation:**

The incidence of amputation and of the most prominent risk factors for amputation, including renal dysfunction and hyperglycaemia, has improved considerably during recent years for people with type 1 diabetes. This finding has important implications for quality of life, health economics and prognosis regarding CVD, indicating a trend shift in the treatment of type 1 diabetes.

**Graphical abstract:**

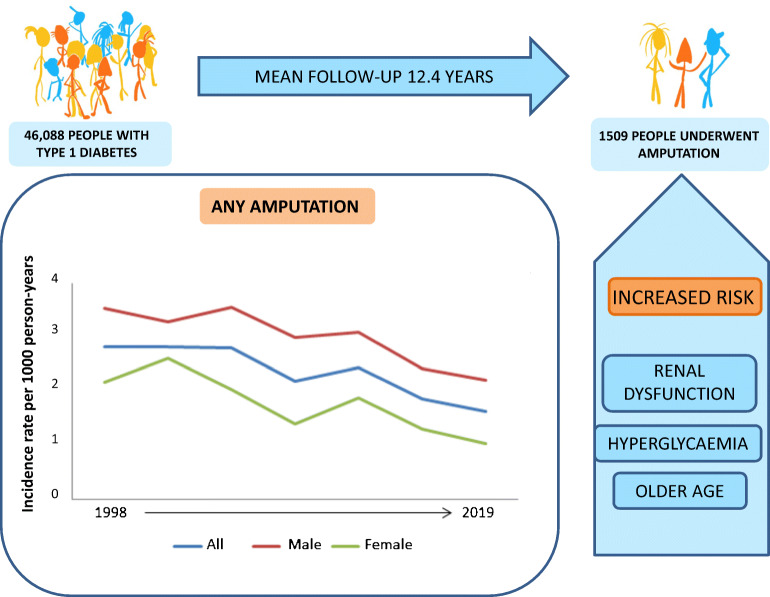

**Supplementary Information:**

The online version contains peer-reviewed but unedited supplementary material available at 10.1007/s00125-021-05550-z.

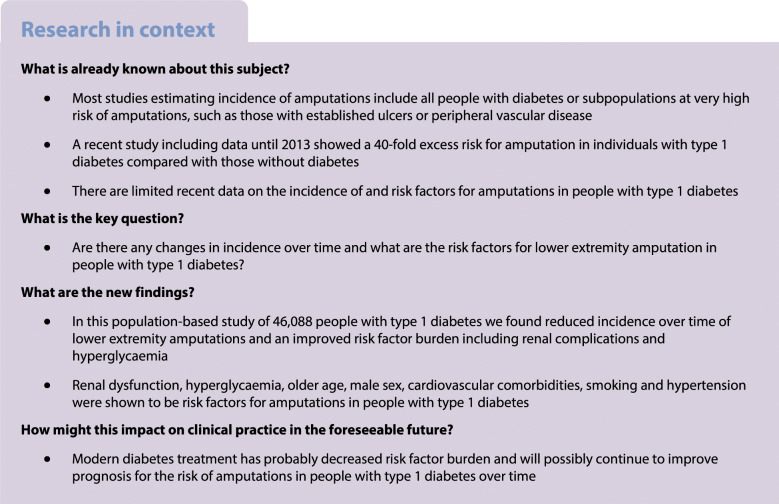



## Introduction

Diabetes foot ulcers are common, healing is often delayed and limb loss through amputation is a not infrequent final outcome [1]. Lower extremity amputation (LEA) in people with diabetes is a major source of disability and distress and constitutes a significant financial burden for the healthcare system [2–4]. About half of all non-traumatic amputations in the western world are attributable to diabetes, and an earlier study showed a 40-fold excess risk of amputations in people with type 1 diabetes compared with the general population [5].

There are multiple pathways to diabetic foot ulceration but the contributions of micro- and macrovascular disease are prominent [6, 7]. Thus, risk factors for these conditions, such as hyperglycaemia, hypertension and hyperlipidaemia, could be related to LEA in type 1 diabetes [1, 6]. Changes in this risk factor burden, improved diabetic foot ulcer care following the introduction of multidisciplinary teams, and extended use of revascularisation have possibly reduced the rate of LEA [8–10].

The risk of LEA has mostly been studied in populations with type 2 diabetes or in mixed groups of individuals with diabetes and findings suggest that LEA is associated with older age, male sex, renal dysfunction and worse glycaemic control [11–16]. Recent studies on incidence and a wide range of risk factors are scarcer in people with type 1 diabetes.

The aim of this study was to investigate incidence over time and potential risk factors, including age, sex, diabetes duration, smoking, BMI, HbA_1c_, BP, lipid profile, renal function, nephropathy and cardiovascular comorbidities, and their associations with LEA in people with type 1 diabetes.

## Methods

### Study cohort

This study is an observational, population-based cohort study of individuals with type 1 diabetes, 18 years of age or older, in Sweden using data from the Swedish National Diabetes Register (NDR). The cohort was linked to several national Swedish health registers through each unique Swedish personal identification number. These included the National Patient Register (NPR), the Swedish Cause of Death Register and the Longitudinal Integration Database for Health Insurance and Labor Market Studies. This study was approved by the regional ethics review board at the University of Gothenburg, Sweden.

The NDR was initiated in 1996 and has been described elsewhere [17]. This register has nationwide coverage and virtually all patients in Sweden with type 1 diabetes are included. The register contains data collected from patient encounters at primary healthcare clinics or hospital outpatient clinics. Collected information includes diabetes-related characteristics and treatment, anthropometrics, risk factors and diabetes-related complications*.* The epidemiological definition of type 1 diabetes, treatment with insulin and diagnosis at age 30 years or younger, has been adapted to define individuals with type 1 diabetes, and this definition has been estimated to be correct in about 97% of cases [18]. All people with type 1 diabetes and with at least one registration in the NDR between 1 January 1998 and 2 October 2019 and no previous amputation were included in the study cohort.

The Longitudinal Integration Database for Health Insurance and Labor Market Studies was used for information on country of birth (Sweden or abroad) and level of education stratified into three groups: (1) low (up to 9 years); (2) intermediate (10–12 years); and (3) high (university or college).

The Swedish NPR contains nationwide hospital discharge information since 1987 and is operated by the Swedish National Board of Health and Welfare. Diagnoses in the NPR are registered according to ICD-9 (http://www.icd9data.com/2007/Volume1/default.htm) and ICD-10 (http://apps.who.int/classifications/icd10/browse/2016/en).

Information on time and cause of death was retrieved from the Swedish Cause of Death Register.

### Outcome

Amputations and comorbidities, including CHD, heart failure, valve disease, stroke, atrial fibrillation and cancer, were collected from the NPR (ICD codes are described in electronic supplementary materials [ESM] Methods section) and mortality data were collected from the Swedish Cause of Death register. Amputation rate was analysed at the anatomical level, minor or major LEA, and at the person level (incidence rate) or limb (first amputation per calendar year).

### Exposures

Diabetes duration, smoking status, systolic BP (SBP), diastolic BP (DBP), HbA_1c_, creatinine level, albuminuria, LDL-cholesterol and HDL-cholesterol were collected from the NDR. Smoking was defined as current or former (no smoking during the last 3 months). The CKD-EPI equation was used to calculate eGFR, and kidney function was classified according to the National Kidney Foundation [19]. Microalbuminuria was defined as at least two positive results within 1 year and defined as an albumin/creatinine ratio of 3–30 mg/mmol (30–300 mg/g) or urinary albumin clearance of 20–200 μg/min (20–300 mg/l). Higher ratios defined macroalbuminuria.

### Statistical analysis

Crude incidence rates were estimated and standardised for age and sex per 1000 person-years. Continuous variables are described using mean ± SD and median (IQR) values. Categorical variables are presented as *n* (%). Poisson regression was used to estimate 95% CIs for event rates per 1000 person-years. Incidence over time of LEA was evaluated over the following periods: 1998–2001; 2002–2004; 2005–2007; 2008–2010; 2011–2013; 2014–2016; and 2017–2019. Incidence of LEA was estimated as the unadjusted incidence overall, by sex and age category, and the standardised incidence for age and sex (according to the distribution of the first time period) over time was also determined.

HRs with 95% CIs, for time to first amputation, time to first major amputation and time to first minor amputation, were estimated with Cox regression models using time-updated means from baseline and onwards (see ESM Methods section). Patients were censored at death or at the date of last data retrieval (2 October 2019). Updated means were calculated for BMI, SBP, DBP, HbA_1c_, LDL-cholesterol, HDL-cholesterol and time-updated values for age, smoking, eGFR, albuminuria categories and comorbidities. Model 1 was adjusted for age and sex. Model 2 was further adjusted for education, birth in Sweden, time-updated diabetes duration and baseline comorbidities (CHD, heart failure, valve disease, atrial fibrillation and stroke). Model 3 was further adjusted for time-updated potential risk factors (smoking, HbA_1c_, SBP and BMI, unless studied as main effect variable). The gradient of risk was estimated as the change in HR per 1 SD increase or decrease as appropriate.

The longitudinal levels of HbA_1c_ and eGFR over calendar years were estimated using mixed models for repeated measures adjusted for age and sex. Calendar year was analysed as factor variable for estimations presented in figures. For the purpose of describing overall linear increase/decrease over time, calendar year was analysed as a linear continuous variable in the model.

For tests between groups, Fisher’s Exact test was used for dichotomous variables and *t* test for continuous variables. The time-updated value of each variable was calculated as the mean value of all preceding values and updated when a new value was registered. The last registered value was used until the endpoint.

All analyses were performed using SAS software version 9.4 (SAS Institute, Cary, NC, USA). All tests were two-tailed and conducted at the 0.05 significance level.

## Results

### Baseline characteristics

Population characteristics of 46,088 individuals with type 1 diabetes included in the study are presented in Table 1. The mean age was 32.5 years (SD 14.5) and 45% were women. The mean duration of diabetes was 17.2 years (SD 14.5), mean HbA_1c_ was 65.9 mmol/mol (SD 17) (8.2% [SD 1.6]) and 14.2% were smokers. During follow-up 1519 (3.3%) participants underwent an amputation, 609 (1.3%) a minor amputation, 585 (1.3%) a major amputation and 325 (0.7%) both minor and major amputations. Participants with any amputations were older (mean 50.1 years [SD 12.4]), had longer duration of diabetes (34.9 years [SD 13]), and included a higher proportion of men (67.1%) and smokers (19.4%). Participants with any amputation presented with increased burden of cardiovascular comorbidities and risk factors for CVDs compared with those with no amputation. A similar distribution of risk factor burden was also noted in the groups with minor and major amputations when analysed separately (data not shown).

**Table 1:** Baseline characteristics of 46,088 people with type 1 diabetes with no previous amputation registered in the NDR 1998–2019

Characteristic	Total (*n*=46,088)	No amputation (*n*=44,569)	Any amputation (*n*=1519)
Sex, *n* (%)			
Female	20,734 (45.0)	20,235 (45.4)	499 (32.9)
Age, years			
Mean ± SD	32.5 ± 14.5	31.9 ± 14.2	50.1 ± 12.4
Median (Q1, Q3)	28.0 (20.0, 42.0)	27.0 (20.0, 40.0)	50.0 (41.0, 59.0)
*n*	46,088	44,569	1519
Age category, *n* (%)			
18 to <35 years	29,473 (63.9)	29,307 (65.8)	166 (10.9)
35 to <50 years	9485 (20.6)	8943 (20.1)	542 (35.7)
50 to <65 years	5599 (12.1)	4984 (11.2)	615 (40.5)
≥65 years	1531 (3.3)	1335 (3.0)	196 (12.9)
Born in Sweden, *n* (%)	41,881 (90.9)	40,442 (90.7)	1439 (94.7)
Education category, *n* (%)			
Low	11,434 (25.9)	10,908 (25.6)	526 (34.9)
Mid	22,817 (51.7)	22,067 (51.8)	750 (49.8)
High	9846 (22.3)	9615 (22.6)	231 (15.3)
Diabetes duration, years			
Mean ± SD	17.2 ± 14.5	16.5 ± 14.2	34.9 ± 13.0
Median (Q1, Q3)	14.0 (6.0, 26.0)	13.0 (6.0, 25.0)	35.0 (26.0, 44.0)
*n*	46,088	44,569	1519
Smoking^a^, *n* (%)	5877 (14.2)	5597 (14.0)	280 (19.4)
BMI, kg/m^2^			
Mean ± SD	25.0 ± 4.3	25.0 ± 4.3	25.6 ± 4.4
Median (Q1, Q3)	24.4 (22.2, 27.1)	24.3 (22.2, 27.0)	25.0 (22.6, 27.8)
*n*	38,305	36,950	1355
HbA_1c_, mmol/mol			
Mean ± SD	65.9 ± 17.0	65.6 ± 16.9	74.3 ± 16.1
Median (Q1, Q3)	64.0 (54.0, 75.0)	64.0 (54.0, 74.0)	73.0 (63.0, 83.0)
*n*	43,747	42,286	1461
HbA_1c_, %			
Mean ± SD	8.18 ± 1.55	8.15 ± 1.55	8.95 ± 1.47
Median (Q1, Q3)	8.01 (7.09, 9.02)	8.01 (7.09, 8.92)	8.83 (7.92, 9.75)
*n*	43,747	42,286	1461
eGFR, ml min^−1^ [1.73 m]^−2^			
Mean ± SD	109.3 ± 25.2	110.1 ± 24.6	73.5 ± 30.4
Median (Q1, Q3)	114.9 (96.3, 127.5)	115.5 (97.1, 127.8)	78.9 (51.2, 95.7)
*n*	22,416	21,953	463
Albuminuria, *n* (%)			
None	29,922 (84.2)	29,289 (85.6)	633 (48.5)
Microalbuminuria	3236 (9.1)	2958 (8.6)	278 (21.3)
Macroalbuminuria	2381 (6.7)	1987 (5.8)	394 (30.2)
LDL (mmol/l)			
Mean ± SD	2.60 ± 0.84	2.60 ± 0.84	2.87 ± 1.02
Median (Q1, Q3)	2.50 (2.02, 3.08)	2.50 (2.02, 3.07)	2.76 (2.18, 3.49)
*n*	18,796	18,415	381
HDL, mmol/l			
Mean ± SD	1.50 ± 0.45	1.50 ± 0.45	1.47 ± 0.48
Median (Q1, Q3)	1.40 (1.20, 1.70)	1.40 (1.20, 1.70)	1.40 (1.10, 1.70)
*n*	19,014	18,630	384
SBP, mmHg			
Mean ± SD	125.3 ± 16.5	124.7 ± 16.1	141.1 ± 19.4
Median (Q1, Q3)	120.0 (115.0, 135.0)	120.0 (115.0, 132.0)	140.0 (130.0, 150.0)
*n*	41,583	40,134	1449
DBP, mmHg			
Mean ± SD	73.3 ± 9.2	73.1 ± 9.1	76.5 ± 10.1
Median (Q1, Q3)	73.0 (70.0, 80.0)	72.0 (69.0, 80.0)	80.0(70.0, 80.0)
*n*	41,484	40,039	1445
CHD (I20-I25), *n* (%)	1804 (3.9)	1520 (3.4)	284 (18.7)
Heart failure (I50), *n* (%)	592 (1.3)	482 (1.1)	110 (7.2)
Valve disease (I05-I09,I34-I36), *n* (%)	223 (0.5)	207 (0.5)	16 (1.1)
Stroke (I61-I64), *n* (%)	633 (1.4)	516 (1.2)	117 (7.7)
Cancer (C00-C97), *n* (%)	776 (1.7)	717 (1.6)	59 (3.9)
Atrial fibrillation (I48), *n* (%)	285 (0.6)	245 (0.5)	40 (2.6)

For cardiovascular comorbidities ICD-10 codes are presented in parentheses

^a^Missing data excluded from analyses

### Changes in risk factors and incidences of LEA over time (1998–2019)

**Fig. 1:**
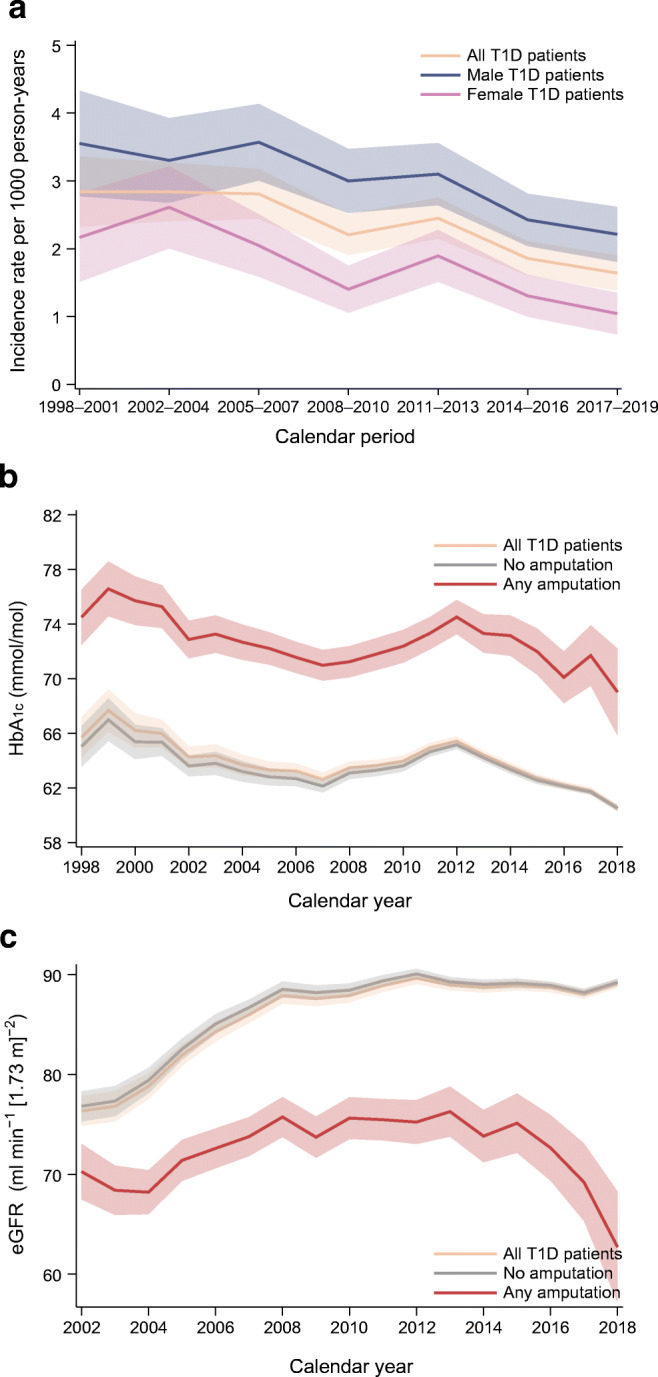
(**a**) Standardised incidence rates with 95% CIs for any amputation in people with type 1 diabetes over time. (**b**) Mean HbA_1c_ with 95% CIs in people with type 1 diabetes and in those with type 1 diabetes and amputation. (**c**) Mean eGFR with 95% CIs in people with type 1 diabetes and in those with type 1 diabetes and amputation. T1D, type 1 diabetes

The crude incidence of any LEA was 2.7 (95% CI 2.6, 2.9) per 1000 person-years during the median follow-up of 12.4 years (IQR 6.6, 18.0) (ESM Table 1). The age- and sex-adjusted incidence of any LEA decreased significantly over time from 1998 to 2019, with a clear drop during the period 2014–2019 (Fig. 1a). The standardised incidence per 1000 person-years was 2.84 (95% CI 2.32, 3.36) in 1998–2001, 2.81 (95% CI 2.44, 3.18) in 2005–2007, 2.45 (95% CI 2.15, 2.75) in 2011–2013 and 1.64 (95% CI 1.38, 1.90) in 2017–2019 (ESM Table 2). During this time, glycaemic control, estimated by HbA_1c_, was significantly worse for patients with LEA than those without LEA and improved over time for those without LEA, with a mean reduction of 0.02% (95% CI 0.02, 0.02) per year (Fig. 1b and ESM Table 3). Renal function, estimated by age- and sex-adjusted eGFR, was worse in participants experiencing LEA and only improved over time for participants without LEA, with a mean improvement of 0.23 (95% CI 0.21, 0.23) ml min^−1^ [1.73 m]^−2^ per year (Fig. 1c and ESM Table 4).

### Effect of potential risk factors on risk for amputation

Table 2 shows results from analyses studying time to amputation through time-updated potential risk factors in the fully adjusted Cox regression model 3. The Cox regression model for time-updated variables for models 1 and 2 are presented in ESM Table 5. Older age was associated with an increased risk of any amputation, HR 1.04 (95% CI 1.03, 1.04; *p* < 0.0001) per year. Moreover, the Cox regression model revealed that significant risk factors for amputations were smoking (HR 1.36 [95% CI 1.17, 1.58]; *p* < 0.0001) and comorbidities including heart failure (HR 3.28 [95% CI 2.86, 3.77]; *p* < 0.0001), CHD (HR 2.26 [95% CI 2.00, 2.55]; *p* < 0.0001), stroke (HR 2.12 [95% CI 1.84, 2.45]; *p* < 0.0001), atrial fibrillation (HR 1.91 [95% CI 1.59, 2.29]; *p* < 0.0001) and valve disease (HR 1.89 [95% CI 1.51, 2.38]; *p* < 0.0001).

**Table 2:** Time-updated potential risk factors and their association to any amputation in people with type 1 diabetes analysed by Cox regression

Variable	No. (%) of events	No. (%) of patients^a^	HR (95% CI)	SD	Standardised HR per 1 SD increase (95% CI)	*p* value
Age	1421 (93.5)	42,270 (91.7)				
Risk by 1 year increase			1.04 (1.03, 1.04)	15.24	1.72 (1.54, 1.92)	<0.0001
Sex	1421 (93.5)	42,270 (91.7)				
Female vs male			0.51 (0.46, 0.57)	0.5	0.72 (0.68, 0.76)	<0.0001
Smoking	1421 (93.5)	42,270 (91.7)				
Risk for yes vs no			1.36 (1.17, 1.58)	0.32	1.10 (1.05, 1.16)	<0.0001
BMI, kg/m^2^	1421 (93.5)	42,270 (91.7)				
Risk by 5 mg/kg^2^ increase			0.92 (0.86, 0.99)	4.02	0.94 (0.89, 0.99)	0.018
HbA_1c_	1421 (93.5)	42,270 (91.7)				
Risk by 10 mmol/mol increase			1.69 (1.63, 1.76)	12.86	1.97 (1.88, 2.06)	<0.0001
Risk by 1% increase			1.78 (1.71, 1.85)	1.18	1.97 (1.88, 2.06)	<0.0001
eGFR,	1228 (80.8)	40,841 (88.6)				
Risk by 10 ml min^−1^ [1.73 m]^−2^ decrease			1.24 (1.21, 1.26)	24.70	1.69 (1.61, 1.77)	<0.0001
Albuminuria category	1397 (92.0)	41,455 (89.9)				
Normoalbuminuria						
Microalbuminuria			1.95 (1.71, 2.23)	0.56	1.46 (1.35, 1.57)	<0.0001
Macroalbuminuria			3.57 (3.12, 4.09)	0.56	2.05 (1.90, 2.21)	<0.0001
LDL-cholesterol, mmol/l	1178 (77.6)	40,235 (87.3)				
Risk by 1 mmol/l increase			0.95 (0.88, 1.04)	0.67	0.97 (0.92, 1.03)	0.27
HDL-cholesterol, mmol/l	1170 (77.0)	40,029 (86.9)				
Risk by 1 mmol/l increase			0.61 (0.52, 0.71)	0.43	0.81 (0.76, 0.86)	<0.0001
SBP, mmHg	1421 (93.5)	42,270 (91.7)				
Risk by 10 mmHg increase			1.28 (1.24, 1.33)	12.84	1.38 (1.31, 1.44)	<0.0001
DBP, mmHg	1420 (93.5)	42,261 (91.7)				
Risk by 5 mmHg increase			1.18 (1.14, 1.23)	6.69	1.25 (1.19, 1.31)	<0.0001
CHD (I20-I25)	1421 (93.5)	42,270 (91.7)				
Risk for yes vs no			2.26 (2.00, 2.55)	0.27	1.25 (1.21, 1.29)	<0.0001
Heart failure (I50)	1421 (93.5)	42,270 (91.7)				
Risk for yes vs no			3.28 (2.86, 3.77)	0.16	1.21 (1.18, 1.23)	<0.0001
Valve disease (I05-I09,I34-I36)	1421 (93.5)	42,270 (91.7)				
Risk for yes vs no			1.89 (1.51, 2.38)	0.11	1.07 (1.04, 1.10)	<0.0001
Atrial fibrillation (I48)	1421 (93.5)	42,270 (91.7)				
Risk for yes vs no			1.91 (1.59, 2.29)	0.13	1.09 (1.06, 1.11)	<0.0001
Stroke (I61-I64)	1421 (93.5)	42,270 (91.7)				
Risk for yes vs no			2.12 (1.84, 2.45)	0.16	1.13 (1.10, 1.16)	<0.0001
Cancer (C00-C97)	1421 (93.5)	42,270 (91.7)				
Risk for yes vs no			1.23 (1.04, 1.45)	0.20	1.04 (1.01, 1.08)	0.018

Table shows risk calculated using model 3, adjusted for time-updated age, sex, education, born in Sweden, time-updated diabetes duration and baseline comorbidities, time-updated variables of smoking, HbA_1c_, SBP, BMI (unless main effect variable)

^a^The analysis for sex and time-updated analyses for age, comorbidities, smoking, HbA_1c_, SBP and BMI included the same patients comprising the complete case population, *n*=42,770. For the analyses of other time-updated variables patients with no follow-up data for the corresponding main effect variable were additionally excluded

**Fig. 2:**
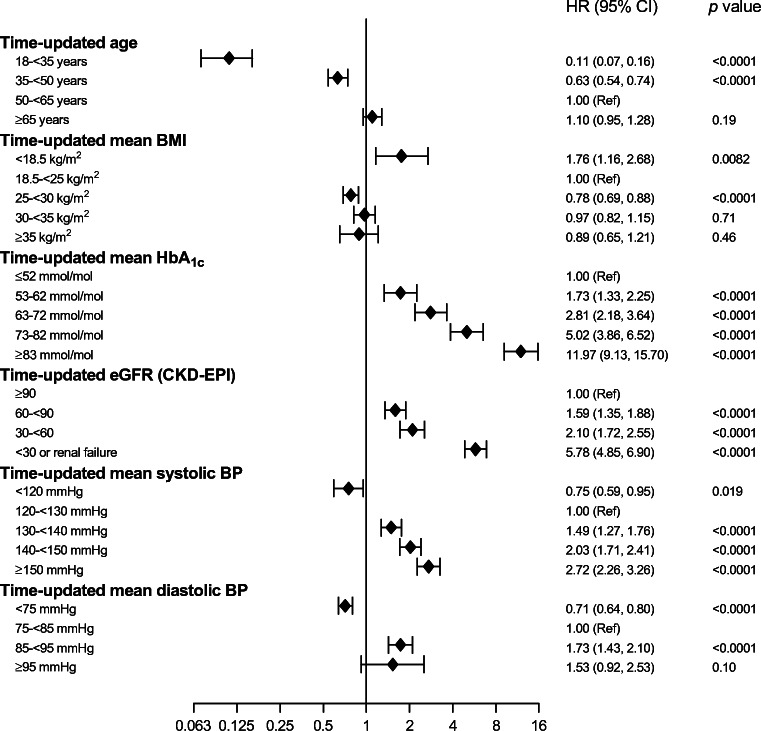
Potential time-updated risk factors by categories and their association with any amputation in people with type 1 diabetes

Higher HbA_1c_ levels were strongly associated with increased risk of amputation in the fully adjusted model, with an HR of 1.69 (95% CI 1.63, 1.76; *p* < 0.0001) per each 10 mmol/mol (~1%) increase. This risk increased monotonically with higher HbA_1c_ and at levels >82 mmol/mol (~9.6%) the HR was 11.97 (95% CI 9.13, 15.70; *p* < 0.0001) (Fig. 2 and ESM Table 6).

Renal dysfunction was associated with increased risk of amputation (HR 1.24 [95% CI 1.21, 1.26], *p* < 0.0001) per 10 ml min^−1^ [1.73 m]^−2^ decrease in eGFR) and related to CKD stages (Table 2, Fig. 2). At eGFR <30 ml min^−1^ [1.73 m]^−2^ the HR was 5.78 (95% CI 4.85, 6.90; *p* < 0.0001) compared with eGFR ≥90 ml min^−1^ [1.73 m]^−2^ in the fully adjusted model.

The risk of amputation was also related to higher BP, with an HR of 1.28 (95% CI 1.24, 1.33; *p* < 0.0001) per each 10 mmHg increase in SBP and 1.18 (95% CI 1.14, 1.23; *p* < 0.0001) per each 5 mmHg increase in DBP. This risk increased monotonically when SBP surpassed 130 mmHg in the fully adjusted model as seen in Fig. 2.

Overall, increasing BMI had a protective effect on the risk of any amputation, with an HR of 0.92 (95% CI 0.86, 0.99; *p* = 0.018) per 5 kg/m^2^ (Table 2). Comparing risk related to different categories of BMI, the risk of amputation associated with being underweight (BMI <18.5 kg/m^2^) was increased while being overweight had a small protective effect and obesity had no significant effect on the risk of any amputation (Fig. 2).

LDL-cholesterol levels were not significantly related to the risk of amputation; however, increasing HDL-cholesterol concentration had a protective effect (HR 0.61 [95% CI 0.52, 0.71]; *p* < 0.0001, per each 1 mmol/l increase) (Table 2).

Similar results were found in the Cox regression models separately analysing minor and major amputations; exceptions were increasing BMI (no protective effect when analysing risk factors for major amputation) and smoking (not significant as a risk factor for minor amputations) (ESM Tables 7, 8).

### Gradient of risk per 1 SD

Gradient of risk per SD was estimated to compare the relative influence of the various risk factors on amputations. The standardised HR per 1 SD was highest for macroalbuminuria, followed by HbA_1c_, age, decreased eGFR, microalbuminuria, male sex, SBP, DBP, cardiovascular comorbidities and smoking (Table 2).

## Discussion

In this nationwide observational cohort study, the risk of LEA was substantially reduced over time and the incidence was more than 40% lower during 2017–2019 than during 1998–2001. The major reduction occurred during 2014–2019 along with clear improvements in glycaemic control and renal function, which were the most prominent risk factors related to LEA.

Older age, male sex, cardiovascular comorbidities, renal dysfunction, increased HbA_1c_, hypertension and smoking were risk factors for amputation in people with type 1 diabetes. There was no statistically significant association between increased LDL-cholesterol concentrations and risk for amputation, while increased HDL concentrations were shown to be protective. The association between risk of amputation and BMI showed no associated monotonic change but being underweight was clearly related to increased risk.

The association between amputation and person-level risk factors in individuals with diabetes has been studied but most studies have been in populations with type 2 diabetes, mixed populations or in very high-risk populations such as people with established foot ulcers [11, 13–15, 20–23]. Sahakyan et al described heavy smoking, hypertension and higher HbA_1c_ to be associated with increased risk of amputation in people with type 1 diabetes [16].

In previous evaluations of incidences of LEA over time no clear reductions were found until 2013, when a 40-fold excess risk compared with the general population was reported [5]. We found that amputation decreased significantly over time, especially from 2014 onwards, along with improved glycaemic control and fewer renal complications. These changes are possibly related to an increased focus on risk factor management, advanced treatments to optimise glycaemic control, and further enhancements in the armamentarium of multidisciplinary diabetes foot clinics, including an increased use of invasive arterial reperfusion.

Diabetes micro- and macroangiopathy are accelerated by local vascular inflammation, hyperlipidaemia, hyperglycaemia and hypertension and lead to ischaemia and loss of protective sensibility, which are risk factors for LEA [1, 6, 7].

In type 1 diabetes, hyperglycaemia is a risk factor for CVD and the association with LEA has been recognised [11, 17, 24, 25]. We found that every 1% increase in HbA_1c_ was associated with a 78% increased risk of amputation. An HbA_1c_ level >72 mmol/mol (8.2%) was associated with a fivefold LEA risk compared with HbA_1c_ <53 mmol/mol (7.0%). The impairment of ulcer healing and acceleration of arteriosclerotic disease may be mechanisms by which hyperglycaemia increase the amputation risk [1, 6, 7].

Interestingly, in our study, amputation rates were lower in the overweight population than in those with normal BMI. This finding might be disturbed by increased risk and more severe comorbidity in those in the lower normal BMI range. This is supported by the significantly higher LEA risk in underweight individuals, a result consistent with earlier studies on BMI and CVD in individuals with type 1 diabetes [26]. Neither obesity nor severe obesity was associated with an increased LEA risk when compared with normal weight.

Hypertension and the closely related diabetes renal dysfunction were both associated with increased risk of amputation. Diabetes renal disease is caused by microangiopathy and usually indicates prolonged antecedent duration of hyperglycaemia solely or in combination with hypertension [27]. Our results support the importance of glycaemic and BP control in order to preserve renal function and reduce the risk of neuropathy and improve healing of diabetic foot ulcers.

Smoking and lower concentrations of HDL-cholesterol increased the risk of amputation, although the impact on risk was minor. However, LDL-cholesterol concentration did not have an effect on the risk of amputation, probably explained by hyperlipidaemia acting indirectly through other LEA risk factors such as renal dysfunction and increased BP.

The current study has several implications since the relatively similar incidence of LEA until 2013 is shown to decrease dramatically during 2014–2019. LEA is a large burden for the affected individual; it is strongly related to reduced quality of life, influences work possibilities for many people and often leads to sick-leave from work [4, 28, 29]. In addition, LEA has a significant impact on immediate and long-term healthcare costs [2]. The improved prognosis for amputations, renal complications and glycaemic control over time indicates a breakthrough in type 1 diabetes care; the prognosis for CVD and mortality will also likely improve, and this will be evaluated in future projects. The strong association between glycaemic control and amputations, as shown in this study, when compared with cardiovascular and renal disease suggests that through significantly improved glycaemic control amputations will probably decrease [17, 24, 25]. Our study indicates a shift in the era of diabetes probably due to the more extensive use of modern equipment in glucose-lowering therapy, such as advanced insulin pumps and continuous glucose monitoring devices [30, 31].

Optimisation of other risk factors, including blood lipids and BP levels, although having less importance than HbA_1c_ as shown here, will further improve prognosis. Generally, there are long time-lags, termed metabolic memory or legacy effects, between optimisation of glycaemic control and complications [32]. Many older diabetic individuals today have had insufficient glucose treatment earlier in life (e.g. only basal insulin therapy with coexisting very high glucose levels). Hence, the current beneficial trends over time seen for LEA will probably be even stronger over the coming years.

This study includes a large number of individuals with type 1 diabetes in Sweden and is based on registers with nationwide coverage of the Swedish population. Data retrieved from the registers also includes information on the most common and updated cardiovascular risk factors, enabling multivariate analysis and estimates on the impact of exposure to each risk factor. The relationship between the risk factors of diabetes micro- and macroangiopathy is complex and therefore analysis of the different potential risk factors for amputations was performed with adjustment for other potential risk factors [6]. The cohort was contemporary, evaluated until 2 October 2019.

Limitations include the lack of information on certain limb-specific risk factors such as neuropathy, reduced circulation, or deformities that affect the risk of amputation. Regression models were created to estimate the risk associated with each risk factor; however, due to the design of this study residual confounding cannot be excluded and a causal relationship between the potential risk factor and outcome cannot be established.

In conclusion, prognosis improved considerably over time during the last decade regarding LEAs in people with type 1 diabetes, while glycaemic control and renal function improved. The strongest risk factors for amputations in people with type 1 diabetes were renal dysfunction and increased HbA_1c_, followed by older age, hypertension, cardiovascular comorbidities and smoking. We found no significant association between LEA and concentrations of LDL-cholesterol but found a protective effect with increased HDL-cholesterol levels. Increased BMI levels are probably not a risk factor for amputation but being underweight was strongly associated with an increased risk of LEAs.

## Supplementary information

ESM (PDF 745 kb)

## Data Availability

The data that support the findings of this study are available from the National Board of Health Welfare in Sweden and from The Swedish NDR. Restrictions apply to the availability of these data, which were used under license for the current study, and according to Swedish legislation are not publicly available.

## Abbreviations

DBP: Diastolic BP
LEA: Lower extremity amputation
NDR: National Diabetes Register
NPR: National Patient Register
SBP: Systolic BP
