# Supplementary material for: Risk factors and incidence over time for lower extremity amputations in people with type 1 diabetes: an observational cohort study of 46,088 patients from the Swedish National Diabetes Registry
Source: Diabetologia. 2021 Sep 8;64(12):2751–61. doi: 10.1007/s00125-021-05550-z (PMC8563633; doi:10.1007/s00125-021-05550-z)
Supplement: Supplementary file 1 — (PDF 745 kb) [file 125_2021_5550_MOESM1_ESM.pdf]

Electronic Supplementary Material to:

Risk factors and incidence over time for lower extremity amputations in people with type 1 diabetes: an observational cohort study of 46 088 patients from the Swedish National Diabetes Registry.

Sara Hallström, Ann-Marie Svensson, Aldina Pivodic, Arndís F. Ólafsdóttir, Magnus Löndahl, Hans Wedel, Marcus Lind

Contents

ESM Methods: ICD codes 3
ESM Methods: SAS code for time-updated Cox regression.....................................3
ESM Table 1. Crude event rates (95% ci) and follow up time for major, minor and any amputations overall and by sex...5
ESM Table 2. Standardized incidence rates with 95% CI per 1000 patient years for LEA, LEA minor, LEA major for diabetes patients, by time-updated calendar periods. 6
ESM Table 3. Longitudinal mean HbA1c all persons and persons with amputation..7
ESM Table 4. Longitudinal mean eGFR all persons and persons with amputation....8
ESM Table 5. Cox proportional hazard models analysing time to any amputation using time-updated variables (n=46 088)..10
ESM Table 6. Cox proportional hazard model 3 analysing time to any amputation using time-updated piecewise linear continuous variables 13
ESM Table 7. Cox proportional hazard models analysing time to major amputation using time-updated variables (n=46 088)..14
ESM Table 8. Cox proportional hazard models analysing time to minor amputation using time-updated variables (n=46 088)..18

ESM Methods: ICD codes

The National patient register (NPR) has a nationwide coverage including the Inpatient register and the Outpatient register. The NPR contains mandatory information on all principal and contributory diagnoses from inpatient and outpatient hospital discharge diagnoses, deaths and causes of death. The following ICD-9 and ICD-10 codes were collected: coronary heart disease 410-414 (ICD-9), I20-I25 (ICD-10) including acute myocardial infarction 410 (ICD-9) and I21 (ICD-10) all main and sub-diagnoses from the inpatient register; stroke 431-434, 436 (ICD-9) and I61-I64 (ICD-10) all main and sub-diagnoses from the inpatient register; hospitalization for heart failure 428 (ICD-9) and I50 (ICD-10) all main and sub-diagnoses from the inpatient register; atrial fibrillation 427 D(ICD-9) and I.48 (ICD-10) all main and sub-diagnoses from both inpatient and outpatient register; cancer 140-208 (ICD-9) and C00-C097 (ICD-10) all main and sub-diagnoses from the inpatient register. For renal failure the diagnose code N185 was used and for renal dialysis the following codes were used DR015, DR016, DR023, DR024, DR013, DR056 all main and sub-diagnoses included from the inpatient register. The ICD-codes related to amputation includes NGQ09, NFQ19 and NFQ99 and diagnosis code Z89.6, Z89.7 (ICD10) for amputations above the knee (mayor). For amputations below knee (mayor) the following ICD-codes are collected NGQ19, NGQ99, NHQ09, NHQ11, for amputations below knee and above ankle (mayor) Z89.5 (ICD10). For minor amputations, below ankle, the following ICD-codes were used NHQ12, NHQ13, NHQ14, NHQ16, NHQ17, NHQ99 and Z89.4 (ICD10).

ESM Methods: SAS code for time-updated Cox regression

```
PROC PHREG DATA = data;
        CLASS mainvar / REF = FIRST;
        MODEL (startyr,stopyr)*endpsplit(0) = mainvar &covars. / RL;
RUN;
```

The dataset data is split up for each patient by consecutive time periods between startyr-stopyr given in years from start of study (first visit date in NDR). For each new value of any variable that is studied as time-updated in the model, there is a new observation for that patient starting with startyr and stopping with stopyr that is either a new updated value or the last updated timepoint ending up in an LEA event or a censoring event. Endpsplit is 0 for all consecutive observations unless it is the last observation, i.e. the last time period, where it is either 0 if censoring or 1 if the studied event has occurred at the end of that time interval (stopyr). Mainvar is for example time-updated HbA1c category with first category corresponding to a reference category. &covars. is a macro-variable including all adjustment variables, e.g. ageupd sex with time-updated age and sex in model A.

SAS software link with an example:
http://support.sas.com/documentation/cdl/en/statug/63033/HTML/default/viewer.htm#statug_phreg_sect038.htm

ESM Table 1. Crude event rates (95% ci) and follow up time for major, minor and any amputations overall and by sex.

Endpoint	Subgroup	n (%) events	Follow-up time (years) Median (IQR)	Crude event rate per 1000 person years (95% CI)*
**Major amputation**	**All**	910 (19.7%)	12.52 (6.66-18.04)	1.6 (1.5 - 1.7)
	**Men**	594 (23.4%)	12.27 (6.53-17.92)	2.0 (1.8 - 2.1)
	**Women**	316 (15.2%)	12.84 (6.86-18.35)	1.2 (1.1 - 1.4)
**Minor amputation**	**All**	934 (20.3%)	12.44 (6.63-18.00)	1.7 (1.6 - 1.8)
	**Men**	658 (26.0%)	12.09 (6.46-17.87)	2.2 (2.0 - 2.4)
	**Women**	276 (13.3%)	12.82 (6.85-18.31)	1.1 (1.0 - 1.2)
**Any amputation**	**All**	1519 (33.0%)	12.37 (6.56-17.96)	2.7 (2.6 - 2.9)
	**Men**	1020 (40.2%)	12.02 (6.36-17.83)	3.4 (3.2 - 3.6)
	**Women**	499 (24.1%)	12.76 (6.80-18.28)	2.0 (1.8 - 2.1)

*95% CI computed by using exact Poisson limits.

ESM Table 2. Standardized incidence rates with 95% CI per 1000 patient years for LEA, LEA minor, LEA major for diabetes patients, by time-updated calendar periods.

Event	Population	1998-2001	2002-2004	2005-2007	2008-2010	2011-2013	2014-2016	2017-2019
**LEA**	**All T1D patients**	2.84 (2.32-3.36)	2.84 (2.40-3.27)	2.81 (2.44-3.18)	2.20 (1.90-2.51)	2.45 (2.15-2.75)	1.86 (1.60-2.11)	1.64 (1.38-1.90)
	**Male T1D patients**	3.55 (2.77-4.33)	3.30 (2.68-3.93)	3.57 (3.00-4.13)	3.00 (2.53-3.47)	3.10 (2.64-3.56)	2.43 (2.04-2.81)	2.21 (1.81-2.62)
	**Female T1D patients**	2.17 (1.51-2.82)	2.61 (2.00-3.21)	2.05 (1.59-2.51)	1.40 (1.05-1.75)	1.89 (1.51-2.28)	1.31 (1.00-1.61)	1.04 (0.74-1.35)
**LEA minor**	**All T1D patients**	1.47 (1.10-1.84)	1.68 (1.34-2.02)	1.72 (1.43-2.02)	1.45 (1.20-1.70)	1.71 (1.45-1.97)	1.15 (0.95-1.36)	0.96 (0.75-1.16)
	**Male T1D patients**	1.94 (1.36-2.51)	2.07 (1.58-2.56)	2.34 (1.88-2.79)	2.02 (1.63-2.41)	2.35 (1.95-2.76)	1.46 (1.16-1.76)	1.41 (1.08-1.73)
	**Female T1D patients**	1.02 (0.57-1.47)	1.50 (1.04-1.96)	1.11 (0.76-1.45)	0.92 (0.63-1.21)	1.05 (0.76-1.34)	0.94 (0.67-1.20)	0.50 (0.28-0.72)
**LEA major**	**All T1D patients**	1.64 (1.25-2.04)	1.65 (1.33-1.98)	1.45 (1.19-1.71)	1.11 (0.90-1.32)	1.27 (1.06-1.48)	1.21 (1.01-1.40)	1.05 (0.84-1.25)
	**Male T1D patients**	1.99 (1.41-2.58)	1.88 (1.42-2.35)	1.75 (1.36-2.13)	1.48 (1.15-1.80)	1.47 (1.18-1.77)	1.55 (1.25-1.85)	1.23 (0.94-1.53)
	**Female T1D patients**	1.29 (0.78-1.79)	1.47 (1.02-1.92)	1.17 (0.83-1.51)	0.71 (0.48-0.95)	1.16 (0.86-1.45)	0.83 (0.59-1.08)	0.83 (0.56-1.10)

ESM Table 3. Longitudinal mean HbA1c all persons and persons with amputation

Group of persons	year	Mean HbA1c (mmol/mol)	95% CI HbA1c (mmol/L)	Mean HbA1c (%)	95% CI HbA1c (%)
All patients	1998	65.7	64.2: 67.2	8.2	8.0: 8.3
All patients	1999	67.7	66.1: 69.2	8.3	8.2: 8.5
All patients	2000	66.2	64.9: 67.5	8.2	8.1: 8.3
All patients	2001	66.0	65.0: 67.0	8.2	8.1: 8.3
All patients	2002	64.3	63.5: 65.0	8.0	8.0: 8.1
All patients	2003	64.3	63.5: 65.2	8.0	8.0: 8.1
All patients	2004	63.7	62.9: 64.4	8.0	7.9: 8.0
All patients	2005	63.3	62.7: 63.9	7.9	7.9: 8.0
All patients	2006	63.3	62.7: 63.8	7.9	7.9: 8.0
All patients	2007	62.6	62.2: 63.1	7.9	7.8: 7.9
All patients	2008	63.5	63.0: 63.9	8.0	7.9: 8.0
All patients	2009	63.6	63.2: 64.1	8.0	7.9: 8.0
All patients	2010	64.0	63.5: 64.4	8.0	8.0: 8.0
All patients	2011	64.9	64.6: 65.3	8.1	8.1: 8.1
All patients	2012	65.4	65.1: 65.8	8.1	8.1: 8.2
All patients	2013	64.4	64.1: 64.7	8.0	8.0: 8.1
All patients	2014	63.5	63.2: 63.8	8.0	7.9: 8.0
All patients	2015	62.7	62.4: 63.0	7.9	7.9: 7.9
All patients	2016	62.2	61.9: 62.5	7.8	7.8: 7.9
All patients	2017	61.8	61.5: 62.0	7.8	7.8: 7.8
All patients	2018	60.5	60.3: 60.8	7.7	7.7: 7.7
All patients	2019	60.0	59.7: 60.3	7.6	7.6: 7.7
No amputation	1998	65.0	63.5: 66.5	8.1	8.0: 8.2
No amputation	1999	67.0	65.4: 68.5	8.3	8.1: 8.4
No amputation	2000	65.4	64.1: 66.6	8.1	8.0: 8.2
No amputation	2001	65.3	64.3: 66.4	8.1	8.0: 8.2
No amputation	2002	63.6	62.8: 64.4	8.0	7.9: 8.0
No amputation	2003	63.8	62.9: 64.6	8.0	7.9: 8.1
No amputation	2004	63.2	62.4: 64.0	7.9	7.9: 8.0
No amputation	2005	62.8	62.2: 63.4	7.9	7.8: 8.0
No amputation	2006	62.7	62.1: 63.2	7.9	7.8: 7.9
No amputation	2007	62.1	61.7: 62.6	7.8	7.8: 7.9
No amputation	2008	63.1	62.6: 63.5	7.9	7.9: 8.0
No amputation	2009	63.3	62.9: 63.7	7.9	7.9: 8.0
No amputation	2010	63.6	63.2: 64.0	8.0	7.9: 8.0
No amputation	2011	64.6	64.3: 65.0	8.1	8.0: 8.1
No amputation	2012	65.2	64.8: 65.5	8.1	8.1: 8.1
No amputation	2013	64.2	63.9: 64.5	8.0	8.0: 8.1
No amputation	2014	63.3	63.0: 63.7	7.9	7.9: 8.0
No amputation	2015	62.6	62.3: 62.9	7.9	7.9: 7.9
No amputation	2016	62.1	61.9: 62.3	7.8	7.8: 7.9
No amputation	2017	61.7	61.5: 61.9	7.8	7.8: 7.8
No amputation	2018	60.5	60.3: 60.7	7.7	7.7: 7.7
No amputation	2019	60.0	59.7: 60.2	7.6	7.6: 7.7
Any amputation	1998	74.5	72.5: 76.5	9.0	8.8: 9.2
Any amputation	1999	76.6	74.6: 78.6	9.2	9.0: 9.3

Group of persons	year	Mean HbA1c (mmol/mol)	95% CI HbA1c (mmol/L)	Mean HbA1c (%)	95% CI HbA1c (%)
Any amputation	2000	75.7	73.9: 77.5	9.1	8.9: 9.2
Any amputation	2001	75.3	73.7: 76.8	9.0	8.9: 9.2
Any amputation	2002	72.9	71.5: 74.2	8.8	8.7: 8.9
Any amputation	2003	73.3	71.9: 74.6	8.9	8.7: 9.0
Any amputation	2004	72.7	71.4: 74.0	8.8	8.7: 8.9
Any amputation	2005	72.2	71.0: 73.4	8.8	8.7: 8.9
Any amputation	2006	71.6	70.4: 72.7	8.7	8.6: 8.8
Any amputation	2007	71.0	69.8: 72.1	8.6	8.5: 8.8
Any amputation	2008	71.2	70.1: 72.4	8.7	8.6: 8.8
Any amputation	2009	71.8	70.6: 73.0	8.7	8.6: 8.8
Any amputation	2010	72.4	71.2: 73.6	8.8	8.7: 8.9
Any amputation	2011	73.3	72.1: 74.5	8.9	8.8: 9.0
Any amputation	2012	74.5	73.3: 75.8	9.0	8.9: 9.1
Any amputation	2013	73.3	71.9: 74.7	8.9	8.7: 9.0
Any amputation	2014	73.1	71.7: 74.6	8.8	8.7: 9.0
Any amputation	2015	72.0	70.3: 73.7	8.7	8.6: 8.9
Any amputation	2016	70.1	68.2: 72.0	8.6	8.4: 8.7
Any amputation	2017	71.7	69.5: 73.9	8.7	8.5: 8.9
Any amputation	2018	69.0	65.8: 72.2	8.5	8.2: 8.8
Any amputation	2019	72.8	64.2: 81.3	8.8	8.0: 9.6

ESM Table 4. Longitudinal mean eGFR all persons and persons with amputation

Group of persons	year	Mean eGFR (ml/min/1.73m^2)	95% CI eGFR (ml/min/1.73m^2)
All patients	2002	76.3	74.8: 77.9
All patients	2003	76.8	75.3: 78.3
All patients	2004	78.8	77.5: 80.1
All patients	2005	81.9	80.9: 83.0
All patients	2006	84.3	83.3: 85.3
All patients	2007	86.0	85.1: 86.8
All patients	2008	87.9	87.1: 88.7
All patients	2009	87.6	86.8: 88.4
All patients	2010	87.9	87.2: 88.6
All patients	2011	88.9	88.3: 89.5
All patients	2012	89.6	89.1: 90.2
All patients	2013	89.0	88.4: 89.5
All patients	2014	88.7	88.2: 89.3
All patients	2015	88.9	88.4: 89.5
All patients	2016	88.7	88.2: 89.2
All patients	2017	88.1	87.6: 88.5
All patients	2018	89.1	88.7: 89.6
All patients	2019	90.2	89.7: 90.7
No amputation	2002	76.8	75.3: 78.3
No amputation	2003	77.3	75.8: 78.9

Group of persons	year	Mean eGFR (ml/min/1.73m^2)	95% CI eGFR (ml/min/1.73m^2)
No amputation	2004	79.4	78.1: 80.7
No amputation	2005	82.5	81.5: 83.6
No amputation	2006	85.1	84.1: 86.1
No amputation	2007	86.7	85.9: 87.5
No amputation	2008	88.5	87.7: 89.3
No amputation	2009	88.2	87.5: 88.9
No amputation	2010	88.4	87.8: 89.1
No amputation	2011	89.4	88.8: 90.0
No amputation	2012	90.1	89.5: 90.6
No amputation	2013	89.3	88.8: 89.8
No amputation	2014	89.0	88.5: 89.5
No amputation	2015	89.2	88.7: 89.6
No amputation	2016	88.9	88.6: 89.3
No amputation	2017	88.2	87.8: 88.6
No amputation	2018	89.2	88.9: 89.6
No amputation	2019	90.2	89.8: 90.6
Any amputation	2002	70.3	67.5: 73.1
Any amputation	2003	68.4	65.9: 70.9
Any amputation	2004	68.2	66.0: 70.4
Any amputation	2005	71.4	69.3: 73.5
Any amputation	2006	72.6	70.6: 74.6
Any amputation	2007	73.8	71.8: 75.7
Any amputation	2008	75.7	73.7: 77.8
Any amputation	2009	73.7	71.7: 75.8
Any amputation	2010	75.6	73.5: 77.7
Any amputation	2011	75.5	73.4: 77.5
Any amputation	2012	75.2	73.0: 77.4
Any amputation	2013	76.3	73.8: 78.8
Any amputation	2014	73.8	71.2: 76.5
Any amputation	2015	75.1	72.1: 78.1
Any amputation	2016	72.7	69.4: 75.9
Any amputation	2017	69.2	65.3: 73.1
Any amputation	2018	62.7	57.2: 68.3
Any amputation	2019	67.4	52.4: 82.3

ESM Table 5. Cox proportional hazard models analysing time to any amputation using time-updated variables (n=46 088)

Model	Number of events	Number of patients	Predictor	Value	Hazard Ratio (95% CI)	SD	Standardized Hazard Ratio per 1 SD increase (95% CI)	P-value
**Model 1**	**1519 (100.0%)**	**46088 (100.0%)**	**Time-updated age (years)**	**Risk by 1 unit increase**	1.08 (1.08 - 1.08)	15.24	3.18 (3.02 - 3.35)	<.0001
**Model 2**	**1507 (99.2%)**	**44097 (95.7%)**	**Time-updated age (years)**		1.03 (1.03 - 1.04)	15.24	1.67 (1.51 - 1.86)	<.0001
**Model 3**	**1421 (93.5%)**	**42270 (91.7%)**	**Time-updated age (years)**		1.04 (1.03 - 1.04)	15.24	1.72 (1.54 - 1.92)	<.0001
**Model 1**	**1519 (100.0%)**	**46088 (100.0%)**	**Sex**	**Men**		0.50		
				**Women**	0.52 (0.47 - 0.58)	0.50	0.73 (0.69 - 0.76)	<.0001
**Model 2**	**1507 (99.2%)**	**44097 (95.7%)**	**Sex**	**Men**		0.50		
				**Women**	0.53 (0.47 - 0.59)	0.50	0.73 (0.69 - 0.77)	<.0001
**Model 3**	**1421 (93.5%)**	**42270 (91.7%)**	**Sex**	**Men**		0.50		
				**Women**	0.51 (0.46 - 0.57)	0.50	0.72 (0.68 - 0.76)	<.0001
**Model 1**	**1491 (98.2%)**	**45275 (98.2%)**	**Time-updated smoking**	**No**		0.32		
				**Yes**	1.68 (1.45 - 1.93)	0.32	1.18 (1.13 - 1.23)	<.0001
**Model 2**	**1479 (97.4%)**	**43521 (94.4%)**	**Time-updated smoking**	**No**		0.32		
				**Yes**	1.65 (1.43 - 1.91)	0.32	1.18 (1.12 - 1.23)	<.0001
**Model 3**	**1421 (93.5%)**	**42270 (91.7%)**	**Time-updated smoking**	**No**		0.32		
				**Yes**	1.36 (1.17 - 1.58)	0.32	1.10 (1.05 - 1.16)	<.0001
**Model 1**	**1459 (96.1%)**	**44348 (96.2%)**	**Time-updated mean BMI (kg/m2)**	**Risk by 5 unit increase**	1.03 (0.97 - 1.10)	4.02	1.02 (0.97 - 1.08)	0.37
**Model 2**	**1449 (95.4%)**	**42737 (92.7%)**	**Time-updated mean BMI (kg/m2)**		1.00 (0.94 - 1.07)	4.02	1.00 (0.95 - 1.06)	0.97
**Model 3**	**1421 (93.5%)**	**42270 (91.7%)**	**Time-updated mean BMI (kg/m2)**		0.92 (0.86 - 0.99)	4.02	0.94 (0.89 - 0.99)	0.018
**Model 1**	**1506 (99.1%)**	**45806 (99.4%)**	**Time-updated mean HbA1c (mmol/mol)**	**Risk by 10 unit increase**	1.71 (1.66 - 1.77)	12.86	2.00 (1.92 - 2.08)	<.0001
**Model 2**	**1494 (98.4%)**	**43944 (95.3%)**	**Time-updated mean HbA1c (mmol/mol)**		1.70 (1.64 - 1.76)	12.86	1.97 (1.89 - 2.06)	<.0001
**Model 3**	**1421 (93.5%)**	**42270 (91.7%)**	**Time-updated mean HbA1c (mmol/mol)**		1.69 (1.63 - 1.76)	12.86	1.97 (1.88 - 2.06)	<.0001
**Model 1**	**1506 (99.1%)**	**45806 (99.4%)**	**Time-updated mean HbA1c (%)**	**Risk by 1 unit increase**	1.80 (1.74 - 1.86)	1.18	2.00 (1.92 - 2.08)	<.0001
**Model 2**	**1494 (98.4%)**	**43944 (95.3%)**	**Time-updated mean HbA1c (%)**		1.78 (1.72 - 1.85)	1.18	1.97 (1.89 - 2.06)	<.0001
**Model 3**	**1421 (93.5%)**	**42270 (91.7%)**	**Time-updated mean HbA1c (%)**		1.78 (1.71 - 1.85)	1.18	1.97 (1.88 - 2.06)	<.0001

Model	Number of events	Number of patients	Predictor	Value	Hazard Ratio (95% CI)	SD	Standardized Hazard Ratio per 1 SD increase (95% CI)	P-value
**Model 1**	**1287 (84.7%)**	**43788 (95.0%)**	**Time-updated eGFR (CKD-EPI)**	**Risk by 10 unit increase**	1.35 (1.33 - 1.38)	24.70	2.11 (2.02 - 2.21)	<.0001
**Model 2**	**1278 (84.1%)**	**42223 (91.6%)**	**Time-updated eGFR (CKD-EPI)**		1.30 (1.27 - 1.32)	24.70	1.91 (1.82 - 2.00)	<.0001
**Model 3**	**1228 (80.8%)**	**40841 (88.6%)**	**Time-updated eGFR (CKD-EPI)**		1.24 (1.21 - 1.26)	24.70	1.69 (1.61 - 1.77)	<.0001
**Model 1**	**1478 (97.3%)**	**44365 (96.3%)**	**Time-updated albuminuria categories**	**None**		0.56		
				**Microalbuminuria (or normal value after microalbuminuria)**	2.89 (2.54 - 3.28)	0.56	1.82 (1.69 - 1.95)	<.0001
				**Macroalbuminuria**	7.00 (6.19 - 7.92)	0.56	2.99 (2.79 - 3.21)	<.0001
**Model 2**	**1466 (96.5%)**	**42756 (92.8%)**	**Time-updated albuminuria categories**	**None**		0.56		
				**Microalbuminuria (or normal value after microalbuminuria)**	2.46 (2.16 - 2.80)	0.56	1.66 (1.54 - 1.79)	<.0001
				**Macroalbuminuria**	5.27 (4.63 - 5.99)	0.56	2.55 (2.37 - 2.74)	<.0001
**Model 3**	**1397 (92.0%)**	**41455 (89.9%)**	**Time-updated albuminuria categories**	**None**		0.56		
				**Microalbuminuria (or normal value after microalbuminuria)**	1.95 (1.71 - 2.23)	0.56	1.46 (1.35 - 1.57)	<.0001
				**Macroalbuminuria**	3.57 (3.12 - 4.09)	0.56	2.05 (1.90 - 2.21)	<.0001
**Model 1**	**1222 (80.4%)**	**42926 (93.1%)**	**Time-updated mean LDL (mmol/L)**	**Risk by 1 unit increase**	1.14 (1.05 - 1.24)	0.67	1.09 (1.03 - 1.16)	0.0020
**Model 2**	**1215 (80.0%)**	**41443 (89.9%)**	**Time-updated mean LDL (mmol/L)**		1.22 (1.13 - 1.33)	0.67	1.15 (1.08 - 1.21)	<.0001
**Model 3**	**1178 (77.6%)**	**40235 (87.3%)**	**Time-updated mean LDL (mmol/L)**		0.95 (0.88 - 1.04)	0.67	0.97 (0.92 - 1.03)	0.27
**Model 1**	**1213 (79.9%)**	**42703 (92.7%)**	**Time-updated mean HDL (mmol/L)**	**Risk by 1 unit increase**	0.43 (0.37 - 0.50)	0.43	0.70 (0.66 - 0.74)	<.0001
**Model 2**	**1205 (79.3%)**	**41238 (89.5%)**	**Time-updated mean HDL (mmol/L)**		0.51 (0.44 - 0.59)	0.43	0.75 (0.71 - 0.80)	<.0001
**Model 3**	**1170 (77.0%)**	**40029 (86.9%)**	**Time-updated mean HDL (mmol/L)**		0.61 (0.52 - 0.71)	0.43	0.81 (0.76 - 0.86)	<.0001
**Model 1**	**1497 (98.6%)**	**45518 (98.8%)**	**Time-updated mean systolic BP (mmHg)**	**Risk by 10 unit increase**	1.35 (1.30 - 1.40)	12.84	1.47 (1.40 - 1.53)	<.0001

Model	Number of events	Number of patients	Predictor	Value	Hazard Ratio (95% CI)	SD	Standardized Hazard Ratio per 1 SD increase (95% CI)	P-value
**Model 2**	**1485 (97.8%)**	**43769 (95.0%)**	**Time-updated mean systolic BP (mmHg)**		1.34 (1.29 - 1.38)	12.84	1.45 (1.39 - 1.52)	<.0001
**Model 3**	**1421 (93.5%)**	**42270 (91.7%)**	**Time-updated mean systolic BP (mmHg)**		1.28 (1.24 - 1.33)	12.84	1.38 (1.31 - 1.44)	<.0001
**Model 1**	**1494 (98.4%)**	**45503 (98.7%)**	**Time-updated mean diastolic BP (mmHg)**	**Risk by 5 unit increase**	1.19 (1.15 - 1.24)	6.69	1.26 (1.21 - 1.33)	<.0001
**Model 2**	**1482 (97.6%)**	**43756 (94.9%)**	**Time-updated mean diastolic BP (mmHg)**		1.24 (1.20 - 1.29)	6.69	1.34 (1.27 - 1.40)	<.0001
**Model 3**	**1420 (93.5%)**	**42261 (91.7%)**	**Time-updated mean diastolic BP (mmHg)**		1.18 (1.14 - 1.23)	6.69	1.25 (1.19 - 1.31)	<.0001
**Model 1**	**1519 (100.0%)**	**46088 (100.0%)**	**Time-updated CHD (I20-I25)**	**No**		0.27		
				**Yes**	3.12 (2.78 - 3.50)	0.27	1.36 (1.32 - 1.41)	<.0001
**Model 2**	**1507 (99.2%)**	**44097 (95.7%)**	**Time-updated CHD (I20-I25)**	**No**		0.27		
				**Yes**	2.35 (2.08 - 2.65)	0.27	1.26 (1.22 - 1.30)	<.0001
**Model 3**	**1421 (93.5%)**	**42270 (91.7%)**	**Time-updated CHD (I20-I25)**	**No**		0.27		
				**Yes**	2.26 (2.00 - 2.55)	0.27	1.25 (1.21 - 1.29)	<.0001
**Model 1**	**1519 (100.0%)**	**46088 (100.0%)**	**Time-updated Heart failure (I50)**	**No**		0.16		
				**Yes**	5.01 (4.42 - 5.68)	0.16	1.29 (1.26 - 1.32)	<.0001
**Model 2**	**1507 (99.2%)**	**44097 (95.7%)**	**Time-updated Heart failure (I50)**	**No**		0.16		
				**Yes**	3.91 (3.42 - 4.48)	0.16	1.24 (1.21 - 1.27)	<.0001
**Model 3**	**1421 (93.5%)**	**42270 (91.7%)**	**Time-updated Heart failure (I50)**	**No**		0.16		
				**Yes**	3.28 (2.86 - 3.77)	0.16	1.21 (1.18 - 1.23)	<.0001
**Model 1**	**1519 (100.0%)**	**46088 (100.0%)**	**Time-updated Valve disease (I05-I09,I34-I36)**	**No**		0.11		
				**Yes**	2.43 (1.95 - 3.02)	0.11	1.10 (1.07 - 1.12)	<.0001
**Model 2**	**1507 (99.2%)**	**44097 (95.7%)**	**Time-updated Valve disease (I05-I09,I34-I36)**	**No**		0.11		
				**Yes**	1.88 (1.50 - 2.35)	0.11	1.07 (1.04 - 1.09)	<.0001
**Model 3**	**1421 (93.5%)**	**42270 (91.7%)**	**Time-updated Valve disease (I05-I09,I34-I36)**	**No**		0.11		
				**Yes**	1.89 (1.51 - 2.38)	0.11	1.07 (1.04 - 1.10)	<.0001
**Model 1**	**1519 (100.0%)**	**46088 (100.0%)**	**Time-updated Atrial fibrillation (I48)**	**No**		0.13		
				**Yes**	2.07 (1.74 - 2.46)	0.13	1.10 (1.07 - 1.12)	<.0001

Model	Number of events	Number of patients	Predictor	Value	Hazard Ratio (95% CI)	SD	Standardized Hazard Ratio per 1 SD increase (95% CI)	P-value
**Model 2**	**1507 (99.2%)**	**44097 (95.7%)**	**Time-updated Atrial fibrillation (I48)**	**No**		0.13		
				**Yes**	1.82 (1.52 - 2.17)	0.13	1.08 (1.06 - 1.11)	<.0001
**Model 3**	**1421 (93.5%)**	**42270 (91.7%)**	**Time-updated Atrial fibrillation (I48)**	**No**		0.13		
				**Yes**	1.91 (1.59 - 2.29)	0.13	1.09 (1.06 - 1.11)	<.0001
**Model 1**	**1519 (100.0%)**	**46088 (100.0%)**	**Time-updated Stroke (I61-I64)**	**No**		0.16		
				**Yes**	2.77 (2.41 - 3.19)	0.16	1.18 (1.15 - 1.21)	<.0001
**Model 2**	**1507 (99.2%)**	**44097 (95.7%)**	**Time-updated Stroke (I61-I64)**	**No**		0.16		
				**Yes**	2.37 (2.06 - 2.72)	0.16	1.15 (1.13 - 1.18)	<.0001
**Model 3**	**1421 (93.5%)**	**42270 (91.7%)**	**Time-updated Stroke (I61-I64)**	**No**		0.16		
				**Yes**	2.12 (1.84 - 2.45)	0.16	1.13 (1.10 - 1.16)	<.0001
**Model 1**	**1519 (100.0%)**	**46088 (100.0%)**	**Time-updated Cancer (C00-C97)**	**No**		0.20		
				**Yes**	1.24 (1.05 - 1.46)	0.20	1.04 (1.01 - 1.08)	0.010
**Model 2**	**1507 (99.2%)**	**44097 (95.7%)**	**Time-updated Cancer (C00-C97)**	**No**		0.20		
				**Yes**	1.14 (0.97 - 1.35)	0.20	1.03 (0.99 - 1.06)	0.11
**Model 3**	**1421 (93.5%)**	**42270 (91.7%)**	**Time-updated Cancer (C00-C97)**	**No**		0.20		
				**Yes**	1.23 (1.04 - 1.45)	0.20	1.04 (1.01 - 1.08)	0.018

Model 1: adjusted for time-updated age and sex.
Model 2: additionally adjusted for eduction, born in Sweden, time-updated diabetes duration and baseline comorbidities.
Model 3: additionally adjusted for time-updated variables of smoking, HbA1c, SBP, BMI (unless main effect variable).

ESM Table 6. Cox proportional hazard model 3 analysing time to any amputation using time-updated piecewise linear continuous variables

			Categories	
**Model**	**Predictor**	**Value**	**Hazard Ratio (95% CI)**	**P-value**
**Model 3**	**Time-updated age**	**18-<35 years**	0.11 (0.07 - 0.16)	<.0001
		**35-<50 years**	0.63 (0.54 - 0.74)	<.0001
		**50-<65 years**		
		**>=65 years**	1.10 (0.95 - 1.28)	0.19
**Model 3**	**Time-updated mean BMI**	**<18.5 kg/m^2**	1.76 (1.16 - 2.68)	0.0082
		**18.5-<25 kg/m^2**		
		**25-<30 kg/m^2**	0.78 (0.69 - 0.88)	<.0001
		**30-<35 kg/m^2**	0.97 (0.82 - 1.15)	0.71
		**>=35 kg/m^2**	0.89 (0.65 - 1.21)	0.46
**Model 3**	**Time-updated mean HbA1c**	**<=6.9% (<=52 mmol/mol)**		

Model	Predictor	Value	Categories<br>Hazard Ratio (95% CI)	P-value
		**7.0-7.8% (53-62 mmol/mol)**	1.73 (1.33 - 2.25)	<.0001
		**7.9-8.7% (63-72 mmol/mol)**	2.81 (2.18 - 3.64)	<.0001
		**8.8-9.6% (73-82 mmol/mol)**	5.02 (3.86 - 6.52)	<.0001
		**>=9.7% (>=83 mmol/mol)**	11.97 (9.13 - 15.70)	<.0001
**Model 3**	**Time-updated eGFR (CKD-EPI)**	**<30 or renal failure**	5.78 (4.85 - 6.90)	<.0001
		**30-<60**	2.10 (1.72 - 2.55)	<.0001
		**60-<90**	1.59 (1.35 - 1.88)	<.0001
		**>=90**		
**Model 3**	**Time-updated mean systolic BP**	**<120 mmHg**	0.75 (0.59 - 0.95)	0.019
		**120-<130 mmHg**		
		**130-<140 mmHg**	1.49 (1.27 - 1.76)	<.0001
		**140-<150 mmHg**	2.03 (1.71 - 2.41)	<.0001
		**>=150 mmHg**	2.72 (2.26 - 3.26)	<.0001
**Model 3**	**Time-updated mean diastolic BP**	**<75 mmHg**	0.71 (0.64 - 0.80)	<.0001
		**75-<85 mmHg**		
		**85-<95 mmHg**	1.73 (1.43 - 2.10)	<.0001
		**>=95 mmHg**	1.53 (0.92 - 2.53)	0.10

Model 3: adjusted for time-updated age and sex, for eduction, born in Sweden, time-updated diabetes duration, baseline comorbidities, time-updated variables of smoking, HbA1c, SBP, BMI (unless main effect variable).

ESM Table 7. Cox proportional hazard models analysing time to major amputation using time-updated variables (n=46 088)

Model	Number of events	Number of patients	Predictor	Value	Hazard Ratio (95% CI)	SD	Standardized Hazard Ratio per 1 SD increase (95% CI)	P-value
**Model 1**	**910 (100.0%)**	**46088 (100.0%)**	**Time-updated age (years)**	**Risk by 1 unit increase**	1.09 (1.08 - 1.09)	15.27	3.68 (3.43 - 3.94)	<.0001
**Model 2**	**902 (99.1%)**	**44097 (95.7%)**	**Time-updated age (years)**		1.04 (1.03 - 1.05)	15.27	1.74 (1.52 - 2.01)	<.0001
**Model 3**	**834 (91.6%)**	**42270 (91.7%)**	**Time-updated age (years)**		1.04 (1.03 - 1.05)	15.27	1.81 (1.56 - 2.10)	<.0001
**Model 1**	**910 (100.0%)**	**46088 (100.0%)**	**Sex**	**Men**		0.50		
				**Women**	0.57 (0.50 - 0.65)	0.50	0.76 (0.71 - 0.81)	<.0001
**Model 2**	**902 (99.1%)**	**44097 (95.7%)**	**Sex**	**Men**		0.50		
				**Women**	0.58 (0.50 - 0.66)	0.50	0.76 (0.71 - 0.81)	<.0001
**Model 3**	**834 (91.6%)**	**42270 (91.7%)**	**Sex**	**Men**		0.50		
				**Women**	0.55 (0.47 - 0.63)	0.50	0.74 (0.69 - 0.80)	<.0001
**Model 1**	**891 (97.9%)**	**45275 (98.2%)**	**Time-updated smoking**	**No**		0.32		

Model	Number of events	Number of patients	Predictor	Value	Hazard Ratio (95% CI)	SD	Standardized Hazard Ratio per 1 SD increase (95% CI)	P-value
				Yes	2.11 (1.77 - 2.51)	0.32	1.27 (1.20 - 1.34)	<.0001
Model 2	883 (97.0%)	43521 (94.4%)	Time-updated smoking	No		0.32		
				Yes	2.06 (1.72 - 2.45)	0.32	1.26 (1.19 - 1.33)	<.0001
Model 3	834 (91.6%)	42270 (91.7%)	Time-updated smoking	No		0.32		
				Yes	1.80 (1.49 - 2.16)	0.32	1.21 (1.14 - 1.28)	<.0001
Model 1	860 (94.5%)	44348 (96.2%)	Time-updated mean BMI (kg/m2)	Risk by 5 unit increase	1.04 (0.96 - 1.13)	4.02	1.03 (0.96 - 1.10)	0.36
Model 2	854 (93.8%)	42737 (92.7%)	Time-updated mean BMI (kg/m2)		1.00 (0.92 - 1.09)	4.02	1.00 (0.93 - 1.07)	1.00
Model 3	834 (91.6%)	42270 (91.7%)	Time-updated mean BMI (kg/m2)		0.95 (0.87 - 1.03)	4.02	0.96 (0.89 - 1.03)	0.23
Model 1	899 (98.8%)	45806 (99.4%)	Time-updated mean HbA1c (mmol/mol)	Risk by 10 unit increase	1.69 (1.62 - 1.77)	12.87	1.97 (1.86 - 2.08)	<.0001
Model 2	891 (97.9%)	43944 (95.3%)	Time-updated mean HbA1c (mmol/mol)		1.67 (1.59 - 1.75)	12.87	1.93 (1.82 - 2.05)	<.0001
Model 3	834 (91.6%)	42270 (91.7%)	Time-updated mean HbA1c (mmol/mol)		1.65 (1.57 - 1.74)	12.87	1.91 (1.79 - 2.04)	<.0001
Model 1	899 (98.8%)	45806 (99.4%)	Time-updated mean HbA1c (%)	Risk by 1 unit increase	1.78 (1.70 - 1.86)	1.18	1.97 (1.86 - 2.08)	<.0001
Model 2	891 (97.9%)	43944 (95.3%)	Time-updated mean HbA1c (%)		1.75 (1.66 - 1.84)	1.18	1.93 (1.82 - 2.05)	<.0001
Model 3	834 (91.6%)	42270 (91.7%)	Time-updated mean HbA1c (%)		1.73 (1.64 - 1.83)	1.18	1.91 (1.79 - 2.04)	<.0001
Model 1	746 (82.0%)	43788 (95.0%)	Time-updated eGFR (CKD-EPI)	Risk by 10 unit increase	1.37 (1.34 - 1.41)	24.78	2.20 (2.07 - 2.34)	<.0001
Model 2	740 (81.3%)	42223 (91.6%)	Time-updated eGFR (CKD-EPI)		1.32 (1.28 - 1.35)	24.78	1.97 (1.85 - 2.10)	<.0001
Model 3	701 (77.0%)	40841 (88.6%)	Time-updated eGFR (CKD-EPI)		1.27 (1.23 - 1.30)	24.78	1.80 (1.68 - 1.92)	<.0001
Model 1	879 (96.6%)	44365 (96.3%)	Time-updated albuminuria categories	None		0.57		
				Microalbuminuria (or normal value after microalbuminuria)	2.44 (2.06 - 2.90)	0.57	1.66 (1.51 - 1.83)	<.0001
				Macroalbuminuria	7.34 (6.27 - 8.58)	0.57	3.09 (2.83 - 3.38)	<.0001
Model 2	871 (95.7%)	42756 (92.8%)	Time-updated albuminuria categories	None		0.57		
				Microalbuminuria (or normal value after microalbuminuria)	1.99 (1.67 - 2.37)	0.57	1.48 (1.34 - 1.63)	<.0001
				Macroalbuminuria	5.32 (4.52 - 6.26)	0.57	2.58 (2.35 - 2.83)	<.0001

Model	Number of events	Number of patients	Predictor	Value	Hazard Ratio (95% CI)	SD	Standardized Hazard Ratio per 1 SD increase (95% CI)	P-value
**Model 3**	**819 (90.0%)**	**41455 (89.9%)**	**Time-updated albuminuria categories**	**None**		0.57		
				**Microalbuminuria (or normal value after microalbuminuria)**	1.58 (1.32 - 1.89)	0.57	1.30 (1.17 - 1.44)	<.0001
				**Macroalbuminuria**	3.78 (3.18 - 4.50)	0.57	2.12 (1.93 - 2.34)	<.0001
**Model 1**	**708 (77.8%)**	**42926 (93.1%)**	**Time-updated mean LDL (mmol/L)**	**Risk by 1 unit increase**	1.21 (1.08 - 1.35)	0.67	1.13 (1.05 - 1.22)	0.0008
**Model 2**	**704 (77.4%)**	**41443 (89.9%)**	**Time-updated mean LDL (mmol/L)**		1.30 (1.17 - 1.45)	0.67	1.19 (1.11 - 1.28)	<.0001
**Model 3**	**673 (74.0%)**	**40235 (87.3%)**	**Time-updated mean LDL (mmol/L)**		0.99 (0.88 - 1.11)	0.67	0.99 (0.92 - 1.07)	0.86
**Model 1**	**700 (76.9%)**	**42703 (92.7%)**	**Time-updated mean HDL (mmol/L)**	**Risk by 1 unit increase**	0.40 (0.33 - 0.48)	0.43	0.67 (0.62 - 0.73)	<.0001
**Model 2**	**695 (76.4%)**	**41238 (89.5%)**	**Time-updated mean HDL (mmol/L)**		0.48 (0.40 - 0.59)	0.43	0.73 (0.68 - 0.80)	<.0001
**Model 3**	**666 (73.2%)**	**40029 (86.9%)**	**Time-updated mean HDL (mmol/L)**		0.58 (0.47 - 0.72)	0.43	0.79 (0.73 - 0.87)	<.0001
**Model 1**	**891 (97.9%)**	**45518 (98.8%)**	**Time-updated mean systolic BP (mmHg)**	**Risk by 10 unit increase**	1.35 (1.29 - 1.41)	12.87	1.47 (1.38 - 1.55)	<.0001
**Model 2**	**883 (97.0%)**	**43769 (95.0%)**	**Time-updated mean systolic BP (mmHg)**		1.33 (1.27 - 1.39)	12.87	1.44 (1.36 - 1.53)	<.0001
**Model 3**	**834 (91.6%)**	**42270 (91.7%)**	**Time-updated mean systolic BP (mmHg)**		1.29 (1.23 - 1.35)	12.87	1.38 (1.30 - 1.47)	<.0001
**Model 1**	**888 (97.6%)**	**45503 (98.7%)**	**Time-updated mean diastolic BP (mmHg)**	**Risk by 5 unit increase**	1.20 (1.14 - 1.26)	6.70	1.28 (1.20 - 1.36)	<.0001
**Model 2**	**880 (96.7%)**	**43756 (94.9%)**	**Time-updated mean diastolic BP (mmHg)**		1.24 (1.19 - 1.31)	6.70	1.34 (1.26 - 1.43)	<.0001
**Model 3**	**833 (91.5%)**	**42261 (91.7%)**	**Time-updated mean diastolic BP (mmHg)**		1.19 (1.14 - 1.25)	6.70	1.27 (1.19 - 1.35)	<.0001
**Model 1**	**910 (100.0%)**	**46088 (100.0%)**	**Time-updated CHD (I20-I25)**	**No**		0.27		
				**Yes**	3.43 (2.97 - 3.97)	0.27	1.40 (1.35 - 1.46)	<.0001
**Model 2**	**902 (99.1%)**	**44097 (95.7%)**	**Time-updated CHD (I20-I25)**	**No**		0.27		
				**Yes**	2.52 (2.16 - 2.93)	0.27	1.29 (1.24 - 1.34)	<.0001
**Model 3**	**834 (91.6%)**	**42270 (91.7%)**	**Time-updated CHD (I20-I25)**	**No**		0.27		

Model	Number of events	Number of patients	Predictor	Value	Hazard Ratio (95% CI)	SD	Standardized Hazard Ratio per 1 SD increase (95% CI)	P-value
				**Yes**	2.47 (2.12 - 2.89)	0.27	1.28 (1.23 - 1.34)	<.0001
**Model 1**	**910 (100.0%)**	**46088 (100.0%)**	**Time-updated Heart failure (I50)**	**No**		0.16		
				**Yes**	5.64 (4.84 - 6.59)	0.16	1.32 (1.29 - 1.35)	<.0001
**Model 2**	**902 (99.1%)**	**44097 (95.7%)**	**Time-updated Heart failure (I50)**	**No**		0.16		
				**Yes**	4.24 (3.60 - 5.00)	0.16	1.26 (1.23 - 1.29)	<.0001
**Model 3**	**844 (92.7%)**	**42543 (92.3%)**	**Time-updated Heart failure (I50)**	**No**		0.16		
				**Yes**	3.61 (3.05 - 4.28)	0.16	1.23 (1.20 - 1.26)	<.0001
**Model 1**	**910 (100.0%)**	**46088 (100.0%)**	**Time-updated Valve disease (I05-I09,I34-I36)**	**No**		0.11		
				**Yes**	2.22 (1.68 - 2.92)	0.11	1.09 (1.06 - 1.12)	<.0001
**Model 2**	**902 (99.1%)**	**44097 (95.7%)**	**Time-updated Valve disease (I05-I09,I34-I36)**	**No**		0.11		
				**Yes**	1.68 (1.27 - 2.23)	0.11	1.06 (1.03 - 1.09)	0.0003
**Model 3**	**834 (91.6%)**	**42270 (91.7%)**	**Time-updated Valve disease (I05-I09,I34-I36)**	**No**		0.11		
				**Yes**	1.71 (1.28 - 2.28)	0.11	1.06 (1.03 - 1.09)	0.0003
**Model 1**	**910 (100.0%)**	**46088 (100.0%)**	**Time-updated Atrial fibrillation (I48)**	**No**		0.13		
				**Yes**	2.28 (1.85 - 2.79)	0.13	1.11 (1.08 - 1.14)	<.0001
**Model 2**	**902 (99.1%)**	**44097 (95.7%)**	**Time-updated Atrial fibrillation (I48)**	**No**		0.13		
				**Yes**	1.98 (1.61 - 2.45)	0.13	1.09 (1.06 - 1.12)	<.0001
**Model 3**	**834 (91.6%)**	**42270 (91.7%)**	**Time-updated Atrial fibrillation (I48)**	**No**		0.13		
				**Yes**	2.09 (1.69 - 2.60)	0.13	1.10 (1.07 - 1.13)	<.0001
**Model 1**	**910 (100.0%)**	**46088 (100.0%)**	**Time-updated Stroke (I61-I64)**	**No**		0.16		
				**Yes**	3.49 (2.96 - 4.11)	0.16	1.23 (1.20 - 1.26)	<.0001
**Model 2**	**902 (99.1%)**	**44097 (95.7%)**	**Time-updated Stroke (I61-I64)**	**No**		0.16		
				**Yes**	2.94 (2.50 - 3.47)	0.16	1.19 (1.16 - 1.23)	<.0001
**Model 3**	**834 (91.6%)**	**42270 (91.7%)**	**Time-updated Stroke (I61-I64)**	**No**		0.16		
				**Yes**	2.63 (2.22 - 3.11)	0.16	1.17 (1.14 - 1.21)	<.0001
**Model 1**	**910 (100.0%)**	**46088 (100.0%)**	**Time-updated Cancer (C00-C97)**	**No**		0.20		
				**Yes**	1.27 (1.04 - 1.55)	0.20	1.05 (1.01 - 1.09)	0.019
**Model 2**	**902 (99.1%)**	**44097 (95.7%)**	**Time-updated Cancer (C00-C97)**	**No**		0.20		
				**Yes**	1.18 (0.96 - 1.44)	0.20	1.03 (0.99 - 1.08)	0.11

Model	Number of events	Number of patients	Predictor	Value	Hazard Ratio (95% CI)	SD	Standardized Hazard Ratio per 1 SD increase (95% CI)	P-value
**Model 3**	**834 (91.6%)**	**42270 (91.7%)**	**Time-updated Cancer (C00-C97)**	**No**		0.20		
				**Yes**	1.22 (0.99 - 1.50)	0.20	1.04 (1.00 - 1.09)	0.059

Model 1: adjusted for time-updated age and sex.
Model 2: additionally adjusted for eduction, born in Sweden, time-updated diabetes duration and baseline comorbidities.
Model 3: additionally adjusted for time-updated variables of smoking, HbA1c, SBP, BMI (unless main effect variable).

ESM Table 8. Cox proportional hazard models analysing time to minor amputation using time-updated variables (n=46 088)

Model	Number of events	Number of patients	Predictor	Value	Hazard Ratio (95% CI)	SD	Standardized Hazard Ratio per 1 SD increase (95% CI)	P-value
**Model 1**	**934 (100.0%)**	**46088 (100.0%)**	**Time-updated age (years)**	**Risk by 1 unit increase**	1.07 (1.06 - 1.07)	15.27	2.79 (2.61 - 2.98)	<.0001
**Model 2**	**928 (99.4%)**	**44097 (95.7%)**	**Time-updated age (years)**		1.03 (1.02 - 1.04)	15.27	1.63 (1.42 - 1.87)	<.0001
**Model 3**	**889 (95.2%)**	**42270 (91.7%)**	**Time-updated age (years)**		1.03 (1.02 - 1.04)	15.27	1.64 (1.42 - 1.89)	<.0001
**Model 1**	**934 (100.0%)**	**46088 (100.0%)**	**Sex**	**Men**		0.50		
				**Women**	0.46 (0.40 - 0.53)	0.50	0.68 (0.63 - 0.73)	<.0001
**Model 2**	**928 (99.4%)**	**44097 (95.7%)**	**Sex**	**Men**		0.50		
				**Women**	0.46 (0.40 - 0.53)	0.50	0.68 (0.63 - 0.73)	<.0001
**Model 3**	**889 (95.2%)**	**42270 (91.7%)**	**Sex**	**Men**		0.50		
				**Women**	0.46 (0.40 - 0.53)	0.50	0.68 (0.63 - 0.73)	<.0001
**Model 1**	**917 (98.2%)**	**45275 (98.2%)**	**Time-updated smoking**	**No**		0.32		
				**Yes**	1.48 (1.22 - 1.78)	0.32	1.13 (1.07 - 1.20)	<.0001
**Model 2**	**911 (97.5%)**	**43521 (94.4%)**	**Time-updated smoking**	**No**		0.32		
				**Yes**	1.48 (1.22 - 1.78)	0.32	1.13 (1.07 - 1.20)	<.0001
**Model 3**	**889 (95.2%)**	**42270 (91.7%)**	**Time-updated smoking**	**No**		0.32		
				**Yes**	1.17 (0.96 - 1.42)	0.32	1.05 (0.99 - 1.12)	0.12
**Model 1**	**911 (97.5%)**	**44348 (96.2%)**	**Time-updated mean BMI (kg/m2)**	**Risk by 5 unit increase**	1.01 (0.93 - 1.10)	4.02	1.01 (0.94 - 1.08)	0.86
**Model 2**	**905 (96.9%)**	**42737 (92.7%)**	**Time-updated mean BMI (kg/m2)**		0.99 (0.91 - 1.07)	4.02	0.99 (0.92 - 1.06)	0.73
**Model 3**	**889 (95.2%)**	**42270 (91.7%)**	**Time-updated mean BMI (kg/m2)**		0.90 (0.82 - 0.98)	4.02	0.92 (0.86 - 0.98)	0.013
**Model 1**	**930 (99.6%)**	**45806 (99.4%)**	**Time-updated mean HbA1c (mmol/mol)**	**Risk by 10 unit increase**	1.75 (1.68 - 1.82)	12.86	2.05 (1.95 - 2.16)	<.0001

Model	Number of events	Number of patients	Predictor	Value	Hazard Ratio (95% CI)	SD	Standardized Hazard Ratio per 1 SD increase (95% CI)	P-value
**Model 2**	**924 (98.9%)**	**43944 (95.3%)**	**Time-updated mean HbA1c (mmol/mol)**		1.73 (1.66 - 1.81)	12.86	2.03 (1.92 - 2.14)	<.0001
**Model 3**	**889 (95.2%)**	**42270 (91.7%)**	**Time-updated mean HbA1c (mmol/mol)**		1.71 (1.64 - 1.79)	12.86	1.99 (1.88 - 2.11)	<.0001
**Model 1**	**930 (99.6%)**	**45806 (99.4%)**	**Time-updated mean HbA1c (%)**	**Risk by 1 unit increase**	1.84 (1.76 - 1.92)	1.18	2.05 (1.95 - 2.16)	<.0001
**Model 2**	**924 (98.9%)**	**43944 (95.3%)**	**Time-updated mean HbA1c (%)**		1.82 (1.74 - 1.91)	1.18	2.03 (1.92 - 2.14)	<.0001
**Model 3**	**889 (95.2%)**	**42270 (91.7%)**	**Time-updated mean HbA1c (%)**		1.80 (1.71 - 1.89)	1.18	1.99 (1.88 - 2.11)	<.0001
**Model 1**	**808 (86.5%)**	**43788 (95.0%)**	**Time-updated eGFR (CKD-EPI)**	**Risk by 10 unit increase**	1.33 (1.30 - 1.37)	24.75	2.04 (1.93 - 2.16)	<.0001
**Model 2**	**804 (86.1%)**	**42223 (91.6%)**	**Time-updated eGFR (CKD-EPI)**		1.29 (1.25 - 1.32)	24.75	1.86 (1.75 - 1.98)	<.0001
**Model 3**	**779 (83.4%)**	**40841 (88.6%)**	**Time-updated eGFR (CKD-EPI)**		1.21 (1.18 - 1.24)	24.75	1.61 (1.51 - 1.71)	<.0001
**Model 1**	**911 (97.5%)**	**44365 (96.3%)**	**Time-updated albuminuria categories**	**None**		0.57		
				**Microalbuminuria (or normal value after microalbuminuria)**	3.14 (2.67 - 3.70)	0.57	1.91 (1.74 - 2.09)	<.0001
				**Macroalbuminuria**	6.74 (5.75 - 7.90)	0.57	2.94 (2.69 - 3.22)	<.0001
**Model 2**	**905 (96.9%)**	**42756 (92.8%)**	**Time-updated albuminuria categories**	**None**		0.57		
				**Microalbuminuria (or normal value after microalbuminuria)**	2.76 (2.34 - 3.25)	0.57	1.77 (1.62 - 1.95)	<.0001
				**Macroalbuminuria**	5.25 (4.45 - 6.19)	0.57	2.55 (2.32 - 2.80)	<.0001
**Model 3**	**874 (93.6%)**	**41455 (89.9%)**	**Time-updated albuminuria categories**	**None**		0.57		
				**Microalbuminuria (or normal value after microalbuminuria)**	2.19 (1.86 - 2.59)	0.57	1.56 (1.42 - 1.71)	<.0001
				**Macroalbuminuria**	3.39 (2.85 - 4.03)	0.57	1.99 (1.81 - 2.20)	<.0001
**Model 1**	**765 (81.9%)**	**42926 (93.1%)**	**Time-updated mean LDL (mmol/L)**	**Risk by 1 unit increase**	1.09 (0.98 - 1.21)	0.67	1.06 (0.98 - 1.14)	0.13
**Model 2**	**762 (81.6%)**	**41443 (89.9%)**	**Time-updated mean LDL (mmol/L)**		1.16 (1.04 - 1.29)	0.67	1.10 (1.03 - 1.19)	0.0063
**Model 3**	**746 (79.9%)**	**40235 (87.3%)**	**Time-updated mean LDL (mmol/L)**		0.91 (0.82 - 1.01)	0.67	0.94 (0.88 - 1.01)	0.091

Model	Number of events	Number of patients	Predictor	Value	Hazard Ratio (95% CI)	SD	Standardized Hazard Ratio per 1 SD increase (95% CI)	P-value
**Model 1**	**757 (81.0%)**	**42703 (92.7%)**	**Time-updated mean HDL (mmol/L)**	**Risk by 1 unit increase**	0.48 (0.40 - 0.58)	0.43	0.73 (0.68 - 0.79)	<.0001
**Model 2**	**754 (80.7%)**	**41238 (89.5%)**	**Time-updated mean HDL (mmol/L)**		0.57 (0.47 - 0.69)	0.43	0.79 (0.73 - 0.85)	<.0001
**Model 3**	**738 (79.0%)**	**40029 (86.9%)**	**Time-updated mean HDL (mmol/L)**		0.66 (0.55 - 0.81)	0.43	0.84 (0.77 - 0.91)	<.0001
**Model 1**	**923 (98.8%)**	**45518 (98.8%)**	**Time-updated mean systolic BP (mmHg)**	**Risk by 10 unit increase**	1.38 (1.32 - 1.44)	12.87	1.51 (1.42 - 1.60)	<.0001
**Model 2**	**917 (98.2%)**	**43769 (95.0%)**	**Time-updated mean systolic BP (mmHg)**		1.36 (1.30 - 1.43)	12.87	1.49 (1.40 - 1.58)	<.0001
**Model 3**	**889 (95.2%)**	**42270 (91.7%)**	**Time-updated mean systolic BP (mmHg)**		1.29 (1.23 - 1.36)	12.87	1.39 (1.31 - 1.48)	<.0001
**Model 1**	**922 (98.7%)**	**45503 (98.7%)**	**Time-updated mean diastolic BP (mmHg)**	**Risk by 5 unit increase**	1.20 (1.15 - 1.26)	6.70	1.28 (1.20 - 1.36)	<.0001
**Model 2**	**916 (98.1%)**	**43756 (94.9%)**	**Time-updated mean diastolic BP (mmHg)**		1.25 (1.19 - 1.31)	6.70	1.35 (1.27 - 1.43)	<.0001
**Model 3**	**888 (95.1%)**	**42261 (91.7%)**	**Time-updated mean diastolic BP (mmHg)**		1.17 (1.11 - 1.23)	6.70	1.23 (1.16 - 1.31)	<.0001
**Model 1**	**934 (100.0%)**	**46088 (100.0%)**	**Time-updated CHD (I20-I25)**	**No**		0.27		
				**Yes**	2.90 (2.50 - 3.37)	0.27	1.34 (1.29 - 1.40)	<.0001
**Model 2**	**928 (99.4%)**	**44097 (95.7%)**	**Time-updated CHD (I20-I25)**	**No**		0.27		
				**Yes**	2.25 (1.93 - 2.64)	0.27	1.25 (1.20 - 1.30)	<.0001
**Model 3**	**889 (95.2%)**	**42270 (91.7%)**	**Time-updated CHD (I20-I25)**	**No**		0.27		
				**Yes**	2.14 (1.83 - 2.51)	0.27	1.23 (1.18 - 1.29)	<.0001
**Model 1**	**934 (100.0%)**	**46088 (100.0%)**	**Time-updated Heart failure (I50)**	**No**		0.16		
				**Yes**	4.36 (3.69 - 5.16)	0.16	1.27 (1.23 - 1.30)	<.0001
**Model 2**	**928 (99.4%)**	**44097 (95.7%)**	**Time-updated Heart failure (I50)**	**No**		0.16		
				**Yes**	3.37 (2.81 - 4.03)	0.16	1.21 (1.18 - 1.25)	<.0001
**Model 3**	**898 (96.1%)**	**42543 (92.3%)**	**Time-updated Heart failure (I50)**	**No**		0.16		
				**Yes**	2.75 (2.29 - 3.29)	0.16	1.18 (1.14 - 1.21)	<.0001
**Model 1**	**934 (100.0%)**	**46088 (100.0%)**	**Time-updated Valve disease (I05-I09,I34-I36)**	**No**		0.11		
				**Yes**	2.28 (1.70 - 3.07)	0.11	1.09 (1.06 - 1.13)	<.0001

Model	Number of events	Number of patients	Predictor	Value	Hazard Ratio (95% CI)	SD	Standardized Hazard Ratio per 1 SD increase (95% CI)	P-value
**Model 2**	**928 (99.4%)**	**44097 (95.7%)**	**Time-updated Valve disease (I05-I09,I34-I36)**	**No**		0.11		
				**Yes**	1.84 (1.36 - 2.47)	0.11	1.07 (1.03 - 1.10)	<.0001
**Model 3**	**889 (95.2%)**	**42270 (91.7%)**	**Time-updated Valve disease (I05-I09,I34-I36)**	**No**		0.11		
				**Yes**	1.90 (1.41 - 2.56)	0.11	1.07 (1.04 - 1.11)	<.0001
**Model 1**	**934 (100.0%)**	**46088 (100.0%)**	**Time-updated Atrial fibrillation (I48)**	**No**		0.13		
				**Yes**	1.93 (1.53 - 2.44)	0.13	1.09 (1.06 - 1.12)	<.0001
**Model 2**	**928 (99.4%)**	**44097 (95.7%)**	**Time-updated Atrial fibrillation (I48)**	**No**		0.13		
				**Yes**	1.77 (1.40 - 2.25)	0.13	1.08 (1.04 - 1.11)	<.0001
**Model 3**	**889 (95.2%)**	**42270 (91.7%)**	**Time-updated Atrial fibrillation (I48)**	**No**		0.13		
				**Yes**	1.77 (1.39 - 2.25)	0.13	1.08 (1.04 - 1.11)	<.0001
**Model 1**	**934 (100.0%)**	**46088 (100.0%)**	**Time-updated Stroke (I61-I64)**	**No**		0.16		
				**Yes**	2.09 (1.71 - 2.55)	0.16	1.13 (1.09 - 1.17)	<.0001
**Model 2**	**928 (99.4%)**	**44097 (95.7%)**	**Time-updated Stroke (I61-I64)**	**No**		0.16		
				**Yes**	1.76 (1.44 - 2.14)	0.16	1.10 (1.06 - 1.13)	<.0001
**Model 3**	**889 (95.2%)**	**42270 (91.7%)**	**Time-updated Stroke (I61-I64)**	**No**		0.16		
				**Yes**	1.50 (1.23 - 1.84)	0.16	1.07 (1.03 - 1.11)	<.0001
**Model 1**	**934 (100.0%)**	**46088 (100.0%)**	**Time-updated Cancer (C00-C97)**	**No**		0.20		
				**Yes**	1.11 (0.89 - 1.39)	0.20	1.02 (0.98 - 1.07)	0.36
**Model 2**	**928 (99.4%)**	**44097 (95.7%)**	**Time-updated Cancer (C00-C97)**	**No**		0.20		
				**Yes**	1.03 (0.82 - 1.29)	0.20	1.01 (0.96 - 1.05)	0.79
**Model 3**	**889 (95.2%)**	**42270 (91.7%)**	**Time-updated Cancer (C00-C97)**	**No**		0.20		
				**Yes**	1.08 (0.86 - 1.36)	0.20	1.02 (0.97 - 1.06)	0.51
